# Adverse childhood experiences and trauma informed care for chiropractors: a call to awareness and action

**DOI:** 10.1186/s12998-023-00503-2

**Published:** 2023-08-14

**Authors:** Kira J. Baca, Stacie A. Salsbury

**Affiliations:** 1 Palmer College of Chiropractic, 1000 Brady St, Davenport, IA 52803 USA; 2 Palmer Center for Chiropractic Research, Palmer College of Chiropractic, 741 Brady St, Davenport, IA 52803 USA

**Keywords:** Adverse childhood experiences, Patient-centered care, Manipulation, chiropractic, Public Health, Model, biopsychosocial, Psychological trauma, Adult survivors of child adverse events

## Abstract

**Background:**

Trauma is an emotional response to distressing events where coping and subsequent recovery are absent. Adverse Childhood Experiences (ACEs) are traumas, occurring before the age of 18 years, such as child abuse or neglect, caregiver instability, and household dysfunction. Sixty-four percent of the U.S. population report experiencing at least one ACE, with over 1 billion children experiencing abuse and neglect annually worldwide. Chronic exposure to stressful circumstances or multiple traumatic events has negative physiologic impacts. Persons who experience 3 or more ACEs in childhood are at greater risk of poor mental health outcomes and may be more likely to engage in high-risk behaviors, predisposing them to long-term health impacts, such as metabolic diseases, anxiety, depression, substance use, and chronic pain. Trauma informed care (TIC) is a recommended approach to healthcare delivery across professions, especially when a trauma history is suspected. This commentary aims to increase awareness of the impact of ACEs on health outcomes and introduce TIC concepts as they may apply to chiropractic care for adults with a history of ACEs.

**Discussion:**

This commentary reviews an introductory model (4R's: realize, recognize, respond, resist re-traumatization) as one TIC framework used by healthcare practitioners. Prior trauma can lessen trust, alter perceptions of physical touch, and hands-on examinations and chiropractic treatments may trigger stress responses. Using TIC after appropriate training, includes referrals to multidisciplinary providers to address trauma-related concerns outside the scope of chiropractic, and screening for ACEs if deemed appropriate. Creating safe spaces, communicating clearly, avoiding victimizing language, explaining procedures, asking for consent before physical contact, and giving patients choice and control in their own care may avoid triggering prior traumas.

**Conclusion:**

Given the high worldwide prevalence of persons experiencing 3 or more ACEs, TIC principles are practical adaptations to chiropractic care for use with many patient populations. As TIC and ACEs are emerging concepts within chiropractic, students and practitioners are encouraged to undertake additional training to better understand these complex and sensitive topics. Exploratory research on the incidence, presentation, and impacts of various trauma types, including ACEs, to support adoption of TIC in chiropractic settings is essential.

## Background

In chiropractic, trauma often refers to an incident leading to acute and sometimes serious physical injury. Similarly damaging, trauma in a psychological context can be defined as an emotional response to a distressing traumatic event (such as abuse, assault, or neglect), which challenges an individual’s sense of justice, safety, and environmental predictability [1]. When exposed to stressful circumstances, an individual can experience that stress and recover (coping), or experience it as trauma (unable to cope) [2]. Exposure to increasing numbers of ACEs increases the risk of mental health illness [1]. For this commentary, ACEs will be used to illustrate the value of trauma-awareness and informed care for chiropractors.

Childhood living environments and family networks play significant roles in shaping an individual’s health throughout their lifespan [3]. ACEs are traumatic life events that occur before age 18 [4]. ACEs include direct experiences, such as child abuse (physical, emotional, verbal, or sexual) and neglect, or indirect experiences, such as parental divorce, mental illness, substance abuse, and/or incarceration [4–8]. ACEs, as originally defined, is only a small subset of all childhood traumas. Bullying, poverty, natural disasters, civil unrest, racism, and medical trauma are also childhood traumas, though not represented in the original ACEs research (Table 1) [4, 5, 8–10]. ACEs are found with greater frequency in higher health risk populations where reduced access to healthcare and socioeconomic deprivation are found [11–13]. ACEs are a recognized public health crisis that necessitate this call to awareness and action by the chiropractic profession [26–30]. ACEs are highly prevalent, with 1 billion children affected annually, worldwide [14]. Sixty-four percent of U.S. adults and children self-report exposure to at least 1 ACE; 15% self-report a history of 4 or more [15, 16]. The populations who self-report 4 or more ACEs are at a significantly higher risk of experiencing mental health illness and associated comorbidities [11, 17].

**Table 1:** Examples of adverse childhood experiences (ACEs) [5, 76]

*Direct child abuse or maltreatment*
Physical Abuse/Assault
Verbal Abuse
Emotional Abuse
Sexual Abuse/Assault/Rape
Neglect
*Exposure to Household Dysfunction*
Domestic Violence
Household Mental Illness/Suicide
Household Substance Abuse
Parental Loss/Divorce
Household Member Incarceration
Other Adverse Childhood Experiences*
Bullying/Peer Rejection
School Violence
War/Terrorism/Civil Unrest
Racism/Discrimination
Poverty/Food Scarcity
Natural Disasters/Displacement/Traumatic Loss
Childhood Illness/Medical Trauma

^*^Recognized ACEs not included in the ACE screening tool

ACE-affected persons may have endured substantial trauma and suffering in childhood and/or adolescence. Individuals with ACEs are often encumbered by physiologic changes that may negatively impact their lifelong health, social and emotional well-being, and lead them to seek healthcare. [4, 11, 17–32] Those who experience at least 3 or more ACEs report higher rates of chronic pain, depression, anxiety, and may be more likely to engage in risky behaviors, including: smoking, heavy drinking, sexual promiscuity, or other such self-endangerment [4, 17, 19, 20, 23, 29, 33]. The Centers for Disease Control (CDC) suggests that at least 5 of the 10 leading causes of death are associated with ACE-driven behavioral changes [15]. Higher rates of chronic pain in ACE-affected populations suggests psychosocial factors behind some chronic pain patients [34, 35]; a population commonly seen in chiropractic clinics [36, 37].

Trauma Informed Care (TIC) is the recommended approach for all healthcare professionals who work with people affected by ACEs, and other traumatic experiences [5, 38–42]. TIC guidelines recommend practitioner-specific knowledge of, and appropriate responses to, individuals with trauma during care using a patient-centered framework [43, 44]. TIC concepts have existed within the medical and psychological literature for decades [7, 39, 45, 46], with more recent research and dissemination into other fields of healthcare [38, 47, 48], including chiropractic adjacent fields [40, 42].

Healthcare fields, including psychology, medicine, and nursing, promote greater awareness of the long-term harms of ACE trauma among their practitioners and advocate for healthcare systems to deliver care in ways to prevent re-traumatization of ACE-affected individuals [4, 5, 38, 39, 49]. Chiropractors rely upon physical touch, such as palpation and spinal manipulation, to assess health status and render treatment to their patients. Persons with a history of ACEs may have negative situational perceptions of unfamiliar physical touch and difficulty with trust during routine clinical care, which is an important consideration for the chiropractic profession [38, 49–53]. By developing a greater understanding of the health and social effects of ACEs, chiropractors may more readily recognize the need for using trauma-informed practices with *all* of their patients given the hands-on nature of chiropractic care modalities. The aim of this commentary is to inform chiropractors of the public health crisis of adverse childhood experiences, and possible safety concerns for patients with a history of ACE trauma in a chiropractic office. TIC requires all healthcare professionals to understand the impacts of trauma and know how to respond to people presenting with trauma-based concerns, which includes referrals of patients to health and social service professionals with specialized training in managing the medical and psychological needs of patients with a history of trauma. In this article, we provide chiropractors with a broader awareness of ACEs (one type of trauma) in adult patients and discuss why chiropractors might consider integrating TIC approaches into chiropractic settings, based upon the evolving research about TIC in the greater healthcare arena. We present pertinent information about TIC using the 4R’s Model as one example of how trauma-informed principles may be applicable in general patient care. We provide an overview of the physiologic effects and health impacts of ACEs and offer practical examples of delivering trauma-informed chiropractic care, including options for additional training, considerations for ACE screening and referral for multidisciplinary care.

## Trauma informed care

This call to action for the chiropractic profession encourages chiropractors to adopt trauma-informed care (TIC) as a standard of practice.

*What is trauma-informed care?* While originating from the psychological literature, the principles of TIC are recognized across most healthcare professions [5, 38, 45, 48, 54]. Guidance on the long-lasting health impacts of trauma released by the Substance Abuse and Mental Health Services Association (SAMHSA) of the U.S. Department of Health and Human Services spurred the development of several TIC models, variously described as the: a) 6 key principles [45], b) 5 key ingredients [49], c) 5 principles [41], and d) 4R’s [5]. Collectively, these models acknowledge that persons with a history of trauma may experience poorer health and social outcomes over their lifetimes and describe ways to interact with those affected persons. All TIC models recognize the unique perspectives of an individual who has experienced trauma, including their perceptions of healthcare delivery. Each model also articulates biopsychosocial approaches to caring for people with a history of trauma, including ACEs [55]. Refer to Appendix A for definitions related to TIC.

*Why might chiropractors consider adopting a TIC approach to healthcare delivery?* Chiropractors are often portal of entry providers in their communities. They serve an important role in healthcare, as well as public health and safety, in diagnosing, triaging, and referring patients when necessary for treatment outside their scope of practice [56–58]. In addition, chiropractors often see patients over long periods of time engendering rapport and trust, which may lead to trauma disclosure [59]. By increasing awareness of TIC principles, chiropractors can incorporate trauma-informed concepts into the delivery of care with all patients using patient-centered approaches to maximize patient comfort and choice, and minimize potentially traumatic experiences.

With trauma-informed chiropractic care, patients could receive appropriate screenings for past trauma or ongoing abuse at the discretion of trained practitioners, which may include the chiropractor or another healthcare provider. Chiropractors could refer patients with unresolved complaints and history of traumatic experiences for additional evaluation within a trusted network of healthcare professionals [58]. In addition, trauma-informed chiropractors could recommend multidisciplinary care that addresses a patient’s unresolved trauma to complement or replace chiropractic care, as indicated. [38, 39, 42, 43, 48] While trauma-informed care is an evolving concept in chiropractic, TIC is well studied, widely adopted, and increasingly integrated across many professions and healthcare settings [8, 38, 48, 49, 60–66]. However, concerns about the potential impacts of trauma screening do exist, such as the limited research on long-term outcomes of asking patients about their trauma history [67, 68]. Thus, chiropractors considering adopting trauma screening as part of their TIC practice are encouraged to complete formal training on TIC as well as ACE screening implementation prior to use in practice. [9, 64, 69]

## The 4R’s model

To introduce the concept of TIC, this commentary uses the 4R’s Model as one framework that might guide care of patients with histories of trauma. The 2014 SAMHSA report, *Concept of Trauma and Guidance for a Trauma-Informed Approach* introduced the 4R’s as a comprehensive model to engage professionals, organizations, and communities in creating cultures and sustaining systems in which trauma-informed care is the standard of practice. ***Realize, Recognize, Respond*** and ***Resist Re-Traumatization*** are the 4 critical components of the 4R’s Model of TIC as originally described:

A program, organization, or system that is trauma-informed ***realizes*** the widespread impact of trauma and understands potential paths for recovery; ***recognizes*** the signs and symptoms of trauma in clients, families, staff, and others involved with the system; and ***responds*** by fully integrating knowledge about trauma into policies, procedures, and practices; and seeks to actively ***resist re*-*traumatization*.** [45]

**Fig. 1:**
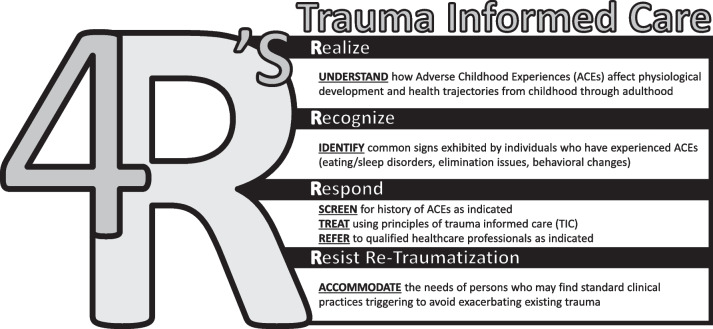
4R’s of Trauma-Informed Care (TIC)–*Visual representation of the components of the 4R’s of TIC* [5, 45]

Figure 1 depicts the 4R’s Model of TIC [5, 45]. While little evidence is available about the use of TIC in chiropractic care settings, research has demonstrated that concepts from the 4R’s model can support the implementation of TIC-informed practice in varied patient populations [5, 70]. This commentary applies the 4R’s Model to adult patients with a history of adverse childhood experiences seeking care in a chiropractic setting. We use ACEs as a window to see into a larger issue that is trauma, its impact, and ways for chiropractors to respond to improve patients’ feelings of safety and security. Chiropractors who identify past or ongoing abuse or neglect in children, adolescents, or dependent adult patients must follow regional laws and regulations regarding any mandatory reporting requirements. The following sections use the 4R’s Model to offer suggestions for implementing TIC in chiropractic practice.

### REALIZE the prevalence and physiological effects of trauma

*TIC emphasizes the importance of REALIZING that many people have endured traumatic experiences, with long-lasting impacts on health, though, recovery is possible* [4, 11, 17, 71]*.* While a large portion of research cited in this paper is based on studies conducted in North America and Australia, statistics from worldwide organizations, such as World Health Organization, illustrate the wide reaching impact of ACEs and other traumas.

ACEs and related traumatic experiences among the global population are common.

- 64% of the United States (U.S.) population reports having experienced ACEs [16]
- 1 billion children worldwide experience abuse or neglect annually [14]
- 75% of children aged 2 to 4 years old receive physical or psychological violence as part of punishment from parents and caregivers [14]
- Childhood sexual abuse affects an estimated 1 in 4 females and 1 in 13 males, with females more prone to physical and sexual abuse than males globally [72, 73]

**Fig. 2:**
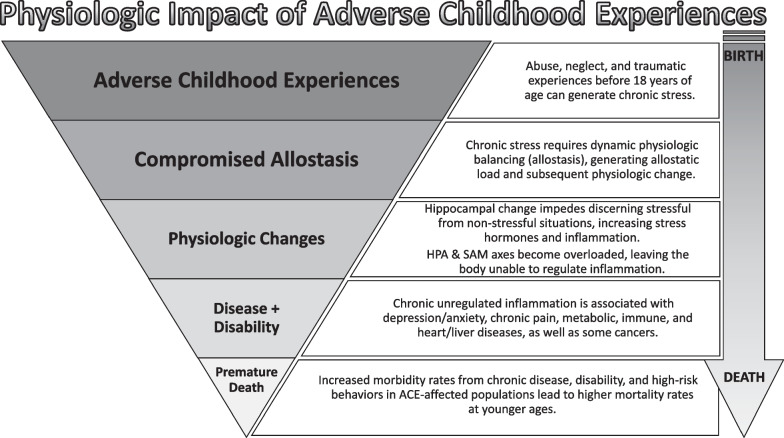
Physiologic Impact of Adverse Childhood Experiences (ACEs) – An inverted pyramid model of physiologic change following ACE exposure over a lifetime. The darkest shaded section represents the total population of ACE-affected individuals and progresses to the lightest, labeled premature death, which affects the lowest percentage of the ACE-affected population relative to the conditions that precede it. [5, 31, 32, 76]. *HPA Axis:* Hypothalamic–Pituitary–Adrenal Axis; SAM Axis: Sympathetic-Adrenal-Medullary axis

*TIC REALIZES the potential physiological effect of trauma on health outcomes and perception of healthcare experiences for individuals with histories of trauma at all stages of life.* Though ACEs can have long-lasting influences on health and wellbeing, resilience against these changes is possible [74, 75]. ACEs can facilitate chronic stress during crucial developmental periods that can negatively influence brain development (Fig. 2) [5, 11, 22, 23, 31, 32, 76, 77]. Chronic stress leads to an increase in stress hormones (adrenocorticoids), held in a dynamic equilibrium by allostasis [31, 78]. Chronic stress generates allostatic load, straining body systems and causing physiological change; the cost of adaptation [32]. Prolonged increase of adrenocorticoids, causes hippocampal hypersensitivity. This sensitivity can lead to interpreting non-threatening situations as threatening, further supporting abnormally high adrenocorticoid levels in a positive feedback loop.

Chronic stress effects the hippocampus by impeding modulators of inflammation, the hypothalamic–pituitary–adrenal (HPA), and the sympathetic-adrenal-medullary (SAM) axes [21, 31]. Heightened chronic inflammatory states are associated with ACE exposure and chronic pain syndromes [34, 35, 79–81]. The biopsychosocial model best captures the link between mental, emotional and physical aspects of pain, and is supported in studies on the impact of early life trauma (ACEs) and chronic adulthood conditions, including chronic pain [4, 19, 28, 82]. Individuals who experience 4 or more types of ACEs are at an increased risk for experiencing poorer health outcomes compared with those reporting 0–3 ACEs [17, 83]. Those with 5 or more ACEs are more likely to have a wider range of negative outcomes than those with fewer ACEs [27]. Metabolic disorders, such as hypertension [84, 85] and diabetes [86–91] are associated with high ACE scores. Similar associations exist with immune disease [11, 31, 92, 93], cancer [24, 25, 76, 94], liver [95] and cardiovascular diseases [18, 96], among other age-related diseases [20]. Some ACEs appear to be more benign than others. While other ACEs appear to have a compounding or synergistic effect on poorer health outcomes following trauma [83]. For example, fibromyalgia has a statistically significant relationship with certain ACEs, including: (1) living in a home with parental mental health issues, (2) physical abuse, (3) emotional abuse, and (4) sexual abuse. [80] Sexual abuse is considered one of the most synergistically reactive traumas, causing increased negative effects when combined with other forms of trauma [83].

However, not all those who experience ACEs report poor health or social outcomes. With the development of positive psychology, researchers began asking about resilience [75]. As a result, research found roughly 50% of those who experienced childhood sexual abuse showed no negative health outcomes [71]. Evidence suggests poor health outcomes following trauma are not absolute and may be mitigated by support networks, learned coping mechanisms, and resiliency mindsets [74, 75, 97]. Persons from populations who experience greater health disparities, such as persons with lower socioeconomic resources [11, 13, 98] or people who have experienced racial or historically driven traumas [10, 99–102] may experience more cumulative or negative effects from ACEs and require additional supports.

### RECOGNIZE signs of trauma and triggered persons

*TIC addresses the importance of RECOGNIZING signs of trauma*. ACE exposure is linked to neural sensitization (chronic pain), depression, anxiety, metabolic illness, autoimmune disease, and high risk behavior [4, 7, 11, 17, 20, 23, 25, 27, 28, 35, 53, 86, 92, 103–107]. Many underlying causes of premature death stem from high risk behaviors involving legal and illicit drug use, dietary choices, sexual promiscuity, and unsafe use of motor vehicles and firearms [27, 108]. Conditions common among people who also report ACEs, include chronic pain [33, 81, 109], headache [110] and fibromyalgia [34, 35, 111, 112], which are problems frequently encountered by chiropractors [113]. Logically, it makes sense that chiropractors who understand ACEs and other traumatic experiences may better recognize affected patients. Then they may choose to adopt trauma-informed practices, such as explaining physical modalities thoroughly prior to performing them to avoid exacerbating prior trauma in their patients [48, 114, 115].

Recognizing trauma and what to do next are all critical aspects of the training offered through programs like AcesAware.org. This example of one organized effort to train healthcare professionals in the state of California, U.S. offers certification that can be tracked on a database of ACE proficient healthcare providers, and is good for practitioners looking for free training and certification in ACE awareness and screening implementation [9, 99]. It is important for practitioners to remember that screening may pose negative ramifications if not delivered responsibly, however there are also significant benefits to screening in the appropriate primary care settings [116]. If ACE screening is deemed unnecessary by the practitioner, it is recommended that TIC principles surrounding recognition and response to trauma still be practiced.

*RECOGNIZING triggers can aid in identifying trauma and improving patient care.* The term trigger refers to internal or external stimuli that elicit a stress response [117, 118]. Stimuli can include touch, sight, sound, smell, taste, emotion, memory, physiologic sensation, or external reminders that cause someone to relive a traumatic event. A trigger may form after exposure to a single or series of traumatic events, though the development of triggers and severity of responses varies among individuals [117–120]. The WHO guidelines on basic training and safety in chiropractic worldwide states that part of chiropractic patient management interventions include psychosocial aspects of patient management [121]. Chiropractors using TIC should seek to identify potentially triggering situations to prevent them from occurring, by generating a safe space for patients to receive care, and gain awareness of aspects of chiropractic care that would potentially trigger ACE-affected individuals. When triggering occurs, individuals experience a resurfacing of traumatic memories or visceral responses. A triggered individual may exhibit fight, flight, or freeze responses to perceived threats, such as overwhelming emotions, involuntary recoil, or shock [122]. Triggered patients may exhibit a change in affect, muscle tension, or guarding posture during physical touch or following a discussion about a sensitive subject. When a patient exhibits signs of triggering, an appropriate response is to stop and ask what caused the reaction. If the person is unable to identify the cause, consider possible responses such as changing patient positioning, the care delivery method, or the number of people in the room to help mitigate triggering effects.

Training both students and practitioners of chiropractic will help increase the profession’s competency and reduce the likelihood of negative incidence related to implementation of trauma screening and subsequent care, and is being implemented in other healthcare professions [65, 123, 124, 124–131]. Outcomes of such training have also begun to be measured, adding credibility to TIC and the addition of ACE awareness in regular practice [46, 62, 64, 125, 128]. For example, a scale was developed and validated to measure Attitudes Related to Trauma-Informed Care (ARTIC) [132].

### RESPOND with training, trauma informed care, screening, and referrals

*Practitioners adopting trauma-informed care RESPOND by training on its principles and practices.* As portal of entry providers in many regions worldwide, it is prudent to consider the implications of not properly identifying and responding to abuse and trauma in patients. TIC will work best when all healthcare professionals adopt patient-centered, trauma-informed practices [82, 133, 134]. Improved outcomes are noted with the implementation of TIC principles in various healthcare settings, although this is yet to be established by research studies within the chiropractic profession [46, 54, 135]. Training in TIC will address expressed deficits in knowledge and is necessary before implementing trauma-informed practices, including the decision to screen patients for a history of ACEs [69, 136].

*TIC models encourage clinicians to RESPOND with safe environments for all patients, reducing opportunities for triggering events.* For example, individuals impacted by ACE trauma are less likely to trust health professionals or have a positive outlook on physical touch [115, 137]. This population may also have more anxiety, negatively influencing executive function involved in making medical decisions [114]. Awareness of the impact of professional touch in healthcare is important, especially when treating patients using physical modalities [138–140]. The high prevalence of physical and sexual abuse among persons with ACEs worldwide suggests that chiropractic patients could be triggered by hands-on modalities, especially treatments or examinations delivered in close physical proximity to the breast, genital, and gluteal regions [138, 139]. Because it is impossible to anticipate all triggering events with certainty, the adoption of universal trauma precautions are recommended [39]. The foundation of universal trauma precaution requires clear communication, confirmation of understanding, frequent requests for consent, and provider flexibility in delivering care (refer to resisting re-traumatization for more information) with all patients. With a universal precautions approach, a practitioner assumes all people have an equal likelihood of having been affected by trauma [39, 45].

*Chiropractors can RESPOND by implementing ACE screening as part of intake procedures *[9]. The ACE screening tool was originally developed for research purposes and tested in two cohort waves. The study had a 68% (18,175/26,824) participation rate in a population of primarily middle class white adults from the Kaiser Permanente’s San Diego Health Appraisal Clinic [4, 141]. Evidence of the effectiveness of ACE screening in chiropractic populations is not known at this time, though some emerging evidence supports the universal use of ACE screening to improve patient care quality and outcomes in other primary care and related healthcare fields [5, 136, 142–145]. Chiropractors assess and identify other psychosocial indicators of abuse [146] making them capable of detecting ACEs with the appropriate training [52]. Training on knowing if, when, and how to screen is crucial when using ACE screening tools [39].

Trauma-informed implementation of screening tools in a clinical setting is a key component of ACE screening. Currently no consensus exists on whether ACE screening should be conducted universally in adults, but is implemented thus in psychological healthcare and pediatric medical populations where identification of childhood trauma can be addressed in real time [5, 38, 39, 136, 143, 143–145]. For further information on when and how to screen patients for ACEs, refer to the reference guide in "Appendix B", and training resources at AcesAware.org [9]. Alternatively, chiropractors may instead encourage patients to share any history of trauma verbally during intake exam or throughout appointments. Patients may also disclose sensitive information regarding abuse or trauma history without prompting or screening, in which case similar guidelines for handling knowledge of their experiences of trauma apply. Such exchanges could require trauma-specific responses. Trauma-specific care goes beyond universal precautions, in that it requires interprofessional collaboration, avoiding practitioners’ own vicarious trauma and triggers, and screening for ACEs if applicable [39].

*RESPOND to knowledge about trauma-affected individuals with referrals for care outside the scope of chiropractic.* Referrals are an extension of professional practice standards in TIC. Referrals separate *universal trauma precautions* from *trauma-specific care *[39]. A key aspect of chiropractic practice is referring patients to other healthcare professionals when appropriate. If screening for trauma, be prepared with a referral network to address the type of problem identified (e.g., sexual abuse hotlines for prior sexual assualt). Referrals for pediatric and adult patients following detection of ACEs or other traumas warrant different types of referrals, in part due to mandatory U.S. reporting requirements, which may also be required in other jurisdictions internationally [5, 39]. Given the diversity of backgrounds and preferences for treatment among chiropractic patients, chiropractors are encouraged to build a similarly diverse referral network of healthcare professionals representative of their patient base. Referral networks should also offer support for populations who experience health disparities due to socio-economic concerns, such as providing free, sliding scale, or low clow-cost services.

The following suggestions support patients in the case of a history of trauma, though they can be implemented universally (see "Appendix B"). If asking patients about trauma, acknowledge a patient’s right to refuse sharing information or decline screening [48]. Where trauma-specific care [39] is required outside the scope of chiropratic practice (ex. psychotherapy), use a well-established professional network for referals, or offer patients a list of professionals to utilize at their own discretion, and based on their own individual needs (insurance, cost, timeframe, location, etc.). Other resources chiropractors can make available to all patients include: (1) publically displayed hotlines and abuse/trauma resource quick response (QR) codes, phone numbers and websites; (2) highly visible and easily accessible brochures and pamphlets on ACEs and other trauma for patient self-edification and (3) business cards of local professionals that may be viewed and taken at patients’ discretion. When these resources are made openly available, patients who do not wish to disclose their personal experiences can find the resources they need, anonymously.

### RESIST RE-TRAUMATIZATION by putting patient concerns first

*Being aware of and acting in accordance with patient concerns are key to RESISTING RE-TRAUMATIZATION of patients.* Resisting re-traumatization involves every aspect of patient care, from the front desk to the treatment room. By implementing training, guidelines, and practice standards, the chiropractic profession can build a culture of trauma awareness, recovery, and healing.

Chiropractors can avoid re-traumatization by creating safe spaces that are comfortable for patients, free of potential triggers, and that provide privacy without isolation. This TIC approach is accomplished through clear communication, avoiding victimizing language, confirming understanding, asking consent before physical contact, and when possible, giving people options for care delivery during appointments. An example in a chiropractic clinical setting includes beginning visits with a conversation about what can be done to make the appointment most comfortable. Specific activities to reduce triggering effects can include, giving thorough explanation and gaining patient consent prior to patient disrobing or examination (if/where appropriate for diagnosis and treatment), using modified exam and treatment positions, using a mirror to see otherwise non-visible procedures, and agreeing to a signal for an acute distress response and/or a wish to stop treatment [39]. Chiropractors should remain vigilant of their proximity to their patient, and any signs that encroachment or physical contact with the patient would be perceived negatively. These measures can improve the quality of care, reduce patients’ fear, and lessen the risk of re-traumatization and potential malpractice litigation [39, 147, 148].

### Chiropractic TIC in Practice: Vignettes

**Fig. 3:**
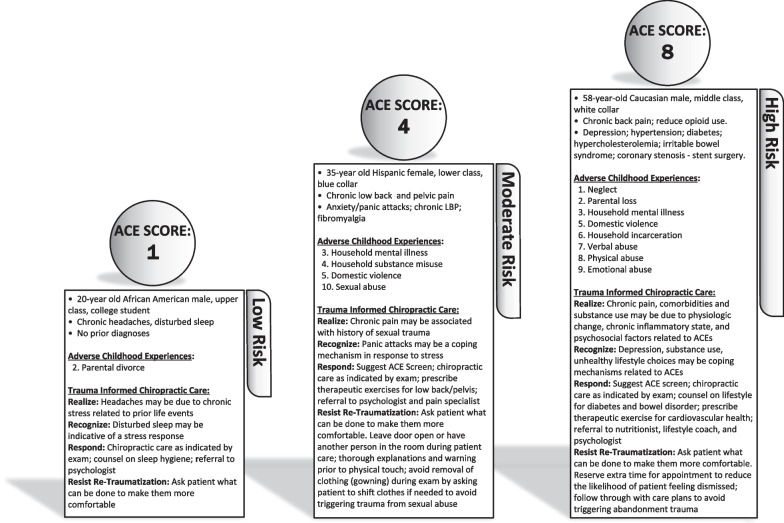
Trauma-Informed Care (TIC) Vignette—Vignettes displaying the clinical application of TIC in 3 hypothetical patients, screened for Adverse childhood experiences (ACEs) using the 4R’s framework with examples

Figure 3 outlines hypothetical examples of patients with varying ACE histories presenting for chiropractic care. In these vignettes, ACE scores are provided, such as would be known when patients were screened for ACEs at intake. Though fictional, these vignettes offer examples of various presentations and TIC-based clinical responses, including co-management with other healthcare providers.

## Discussion

Trauma informed care models illuminate ways of caring for those who have experienced trauma, including ACEs. Keep in mind, ACEs are only one of many types of traumas that may affect your patients, currently or earlier in life. Each type of trauma may present in patients differently, and further training on various types of traumas (intimate partner violence, PTSD, rape, etc.) is suggested to identify these in your patients. As healthcare providers, chiropractors share an ethical obligation with other providers to support patients who have experienced trauma through appropriate identification, treatment, and referral [58, 59]. Whilst ACE-awareness and TIC are emerging topics in chiropractic, awareness and application with chiropractic patients impacted by trauma are aligned with recent efforts within the profession to increase awareness of suicide detection, and recognition and referral for intimate partner violence [146, 149, 150]. Our aim is not that chiropractors perform the psychological aspects of their ACE-affected patients' care, but to *realize*, *recognize*, and *respond* to their patients who do have these needs, without *re-traumatization*. Research to inform TIC practices in different healthcare settings, including chiropractic, are necessary. Chiropractors will require additional training to identify psychosocial factors impacting their patients and to recognized the signs of abuse or trauma [52, 149, 150].

For U.S. chiropractors, SAMHSA guidelines recommend structural changes in 10 domains of healthcare organizations to support TIC, emphasizing implementation at the (1) Governance/Leadership and (2) Policy levels [45]. SAMHSA outlines subsequent levels of integration as (3) Physical Environment, (4) Engagement and Involvement, (5) Cross Sector Collaboration, (6) Screening, Assessment, and Treatment Services, (7) Training and Workforce Development, (8) Progress Monitoring and Quality Assurance, (9) Financing, and (10) Evaluation of Skill [45]. Within these guidelines, domains 3–10 fall upon the chiropractor to implement within a clinical practice, and guidance as to how to implement such practicewide changes are largely absent from the literature. Integration of TIC profession-wide will require involvement of regulatory bodies, educational institutions, health systems, practitioners, and support staff. As noted in a recent scoping review on ACEs, adopting TIC should be done in ways that are congruent with the evolving evidence, and in ways that prevent re-traumatization of patients, are acceptable to practitioners, and do not overburden clinical practices or health systems [68, 71].

Yet, ACEs, and trauma more broadly, are well-established contributors to many behavioral patterns and physiologic maladies that lead to health complications over a lifespan [4, 17, 19]. Promising areas for future research include studying how resilience training may impact health and social outcomes among persons with ACEs and related traumas, and understanding those seemingly unaffected by their trauma [97]. Trainings aimed at mitigating negative effects of screening are available and measures outlined therein should be in place prior to screening. These trainings, among others, are currently available to all practitioners [9]. Discussed previously, triggering patients or practitioners is a potential unintended consequence of trauma screening and an important consideration when deciding whether to implement it into practice [97]. Some concerns that have been raised against the implementation of screening and principles of TIC implementation are: the triggering nature of trauma screening, confidentiality of collected screening data, effects on the patient-doctor relationship, focusing too much on the negative effects of trauma as opposed to reinforcing peoples' resilience, lack of evidence based information on how to deal with trauma-affected individuals after screening [97].

The original ACE study results were based on a primarily white suburban middle-class population, thereby reducing the global generalizability of the results [4, 141]. Also, given the retrospective nature of the data gathering, and potential misinterpretation of what constitutes “abuse” by participants, the ACE study was likely reporting conservatively on the portion of the population affected by ACEs and long term health effects [151]. The original ACE study brought attention to child abuse and household dysfunction, and all screening results reflect that small subset of all potential childhood traumas. The higher prevalence of ACEs in socioeconomically deprived and minority populations, and the traumas that exist therein, are not represented in ACE screening or the original study, potentially making the white middle-class population originally studied a conservative representation of overall ACE-affectedness [2, 11, 152]. The feasibility, acceptability, and satisfaction with ACE screening in chiropractic is not known. Negative unintended outcomes of trauma screening may be possible. It is at the discretion of the chiropractor if or when screening is appropriate, and only after sufficient training in chiropractic schools or accredited training programs, and exposure to literature on TIC and handling of ACE screening reported in other healthcare professions [9, 116]. The potential exists that ACE screening will not be appropriate in all chiropractic offices and settings. In cases of family-practice chiropractors, working extensively with children and populations known to experience greater levels of trauma, it may be more appropriate to screen to gain a better understanding of chronic health conditions than in other types of practices. An ACE score may provide a chiropractor with information to improve care for ACE-affected individuals where psychosocial factors are a major contributor to their condition or perpetuation of chronic pain. This too, is presumed based on discussion and application of ACE screening in other healthcare fields [116, 136, 142–145]. Patients with chronic pain disorders, including fibromyalgia, are often seen in chiropractic offices, and the condition has been associated with psychosocial causes, improving with psychological therapies. [80, 111, 112] In this example, identifying ACEs in connection with fibromyalgia would indicate a referral to a psychological professional.

Increasing awareness of ACEs and TIC in chiropractic education and practice is a first step in addressing these public health and safety concerns within the chiropractic profession. Suggestions for the future of trauma-informed chiropractic care are to: (1) include trauma informed care and other facets of patient-centered care in licensure training, through professional development and seminars from approved by accrediting bodies; (2) develop TIC guidelines and health organization policy and practices based on the experience and success of other healthcare professions; (3) conduct research to better understand TIC application; (4) conduct qualitative research on the perception of chiropractic care by ACE-affected individuals and provider perspectives on barriers and facilitators of TIC; and (5) develop interprofessional networks and attend interdisciplinary workshops to integrate TIC into chiropractic from the experience of other professions.

Furthermore, it falls to practitioners to recognize potential trauma responses of their own [153], and prevent those responses from interfering with patient care. Practitioner trauma responses, including compassion fatigue, can include professional burnout and vicarious traumatization [39, 49]. In these instances, chiropractors are encouraged seek support from the appropriate professionals to address their own unresolved trauma. Whether a chiropractor decides to screen patients for trauma or not, they must be a steward of patients’ experiences, but avoid counseling, commiserating, or consoling them [39].

## Limitations

The evolving nature of TIC research, and the modest number of interventional studies about ACEs, means our suggestions in this paper, while evidence-based, are primarily at the promising and supported levels of quality, rather than strongly supported by systematic reviews of well-designed, randomized controlled trials. While evidence exists in other healthcare fields, the chiropractic profession is in the beginning stages of studying TIC principles in clinical education and practice. Further research about the prevalence and presentation of trauma history among chiropractic patients may be warranted. In addition, differences in scope of practice across various jurisdictions may limit the extent to which individual chiropractors might apply TIC in their clinical practices. Research exploring any negative effects to patients from trauma screening is also needed, as well as whether patients follow through on referral recommendations.

Much of the research on ACEs and TIC were conducted in North American populations, limiting the generalizability of those results worldwide. Furthermore, the ACE-10 item scale was developed for research purposes, rather than for clinical use, suggesting the need for further testing, including in a clinical setting. That same 10-item screening only covers traumas of direct abuse and household dysfunction which excludes significant adversities children face resulting from poverty, racism, and weak social supports. The original ACE study was done on one middle-class Caucasian US-based population and may not be generalizable worldwide, and contains implicit sampling biases [152].

## Conclusion

The ability to identify and respond to ACE-affected patients with trauma-informed care is a valuable skill for all healthcare professionals, including chiropractors. The 4Rs Model of TIC—*Realize, Recognize, Respond* and *Resist Re-Traumatization –* encourages chiropractors to understand the prevalence and effects of ACEs, identify health conditions and behaviors associated with trauma, and generate safe and positive therapeutic experiences for patients. Creating and maintaining an environment of safety, trust, and transparency is an important component of TIC. Screening for ACEs can help identify a person’s history with ACEs, though may not be appropriate for all patients. If opting to screen patients, the proper training and referral network are required.

## Data Availability

Not applicable.

## Appendix A

**Table:**

Term	Definition
Abuse	Interactions in which one person behaves in a cruel, violent, demeaning, or invasive manner toward another person. The term implies physical mistreatment but also encompasses sexual, psychological, or emotional mistreatment.*
Adverse Childhood Experiences (ACEs)	Abuse, neglect, and other traumatic experiences occurring in ages 18 and under. (Goddard, 2021)
Allostasis	The idea that parameters of most physiological regulatory systems change to accommodate environmental demands.*
Allostatic Load	The toll exacted on the body after chronic or repeated activation of the physiological systems that control allostasis in response to life’s challenges.*
Compassion Fatigue	Burnout and stress-related symptoms experienced by healthcare providers, family caregivers, and helping professionals in reaction to interacting with traumatized people over an extended period of time.*
Health Risk Behaviors	High risk behavioral patterns putting an individual to increased risk for disease or injury, and potentially leading to disability, social problems, or death. These behaviors may cluster into a risky lifestyle, such as tobacco, alcohol, or substance use; eating disorders; risky sexual behaviors; and interpersonal violence **High Risk Behaviors**
Neglect	Failure to provide for the basic needs of a person in one’s care. Neglect may be emotional (rejection or apathy), material (withholding food or clothing), or service-oriented (depriving of education or medical attention)*
Trauma	An emotional response to a traumatic event that presents with immediate effects, such as shock and denial, and long-term effects, including unpredictable emotions, flashbacks, strained relationships, and physical symptoms (headaches, nausea).*
Trauma-Informed Care (TIC)	A strengths-based framework grounded in an understanding of and responsiveness to the impact of trauma, emphasizing physical, psychological, and emotional safety for providers and trauma-affected individuals. TIC creates opportunities to rebuild a sense of control and empowerment. (Hopper, Bassuk, & Oliver, 2010, p. 82)
Trauma-Specific Care	Patient-centered care for individuals with a known history of trauma that includes interprofessional collaboration and referral, understanding practitioner’s own history and reactions, and trauma screening. (Raja 2015)
Traumatic Event	Events caused by human behavior (e.g., child abuse, rape, war, industrial accidents) or nature (e.g., earthquakes) that challenge an individual’s sense of justice, safety, and environmental predictability.*
Trigger	A stimulus or reminder of a past trauma that elicits a reaction or a state of emotional arousal.*
Universal Trauma Precautions	Patient-centered care and communication that recognizes the impacts of trauma, reduces patient anxiety, and increases trust/rapport, for use with all patients without screening or knowledge of the individual’s trauma history. (Raja 2015)
Vicarious (Secondary) Traumatization	Provider response from repeated emotionally intimate contact with persons with history of trauma, resulting in changes in the provider’s own worldview and sense of justness and safety.*
*American Psychological Association Dictionary of Psychology	

## Appendix B

**Trauma-Informed Adverse Childhood Experiences (ACEs) Screening:**

Quick Reference Guide.

**Considerations Prior to ACE Screening:**

When screening adult patients for potentially triggering information about ACEs, consider these questions.

1. Can you be a steward of trauma, resisting vicarious victimization and professional burnout from patients divulging difficult emotions and experiences?
2. Do you have a vetted and reliable network of professionals to refer your patients to for services outside the scope of chiropractic?
3. How will an ACE score inform care delivery and potentially impact patient outcomes?

**Table:**

Contents of ACE Screening Tool #	ACE category	Survey question*
1	Neglect	Did you feel you didn’t have enough to eat, had to wear dirty clothes, or had no one to protect or take care of you?
2	Parental Loss/Divorce	Did you lose a parent through divorce, abandonment, death, or other reasons?
3	Household Mental Illness	Did you live with anyone who was depressed, mentally ill, or attempted suicide?
4	Household Substance Abuse	Did you live with anyone who had a problem with drinking/using drugs, including prescription drugs?
5	Domestic Violence	Did your parents or adults in your home ever hit, punch, beat, or threaten to harm each other?
6	Household Incarceration	Did you live with anyone who went to jail or prison?
7	Verbal Abuse	Did a parent or adult in your home ever swear at you, insult you, or put you down?
8	Physical Abuse	Did a parent or adult in your home ever hit, beat, kick, or physically hurt you in any way?
9	Emotional Abuse	Did you feel that no one in your family loved you or thought you were special?
10	Sexual Abuse	Did you experience unwanted sexual contact (such as fondling or oral/anal/vaginal intercourse/penetration)?

*Survey questions based on ACE screening tool for adults through AcesAware.org

**How to use ACE screening tool:**

Screening provides an “ACE Score”. The ACE Score is cumulative as it tallies how many different categories of ACE traumas a person experienced in childhood (above). The ACEs screening tool records self-reported abuse and household dysfunction encountered between the ages of 0–18 years in the form of abuse/maltreatment, exposures, and household dysfunctions. The survey consists of 10 questions, with a maximum score of 10. Each question is worth one point and represents categories of childhood trauma. A score of 4 or greater is considered significant, with higher scores related to poorer health outcomes in adulthood.

Those with > 5 ACEs were reportedly 8 times more likely to have depression and 4 times more likely to have anxiety than those without ACEs. Metabolic disorders, such as hypertension and diabetes also are linked to high ACE scores. Similar associations are noted between elevated ACE scores and immune disease, cancer, liver and cardiovascular disease, and other age-related diseases. There is also evidence that suggests synergistic effects between specific ACEs, leading to increased likelihood of poorer health outcomes.

ACE screening at intake helps understand health outcomes associated with ACEs but generates the need for referral networks to professionals who help individuals with their traumas once reported.

**What to do following ACE screening:**

*Trauma-specific measures*:

- Professionalism
  - Mandatory reporting (U.S. Guideline)
    - Have contacts for Government agencies (U.S. Health and Human Service)
    - Determined on a regional basis and the nature and timeframe of the abuse.
  - Remain within the scope of practice for chiropractors (Determined by location)
    - Utilize a referral network for care outside the scope of chiropractic care
  - Respect patient's right to refuse sharing information/declining screening
- Chiropractor professional referral network:
  - Detailed network of professioanls for specific referrals based on patient care needs and ACE screening results
    - ex. Psychotherapy
  - Provide resources to patients in their appointments to maintain confidentiality

Universal Measures:

- Brochures/pamphlets on abuse, trauma, or ACE related topics
  - Easily accessible, and in plain sight
  - Content appropriate for patient self-education
- Hotlines and local resources:
  - Phone numbers, websites or QR codes
  - Posted in areas patients may access them out of sight from possible abusers
    - Ex. rest rooms
- Business cards of local professionals
  - Ex. counseling services
  - Made available for patients to take anonymously

## Abbreviations

ACEs: Adverse childhood experiences
CDC: Centers for disease control
HPA Axis: Hypothalamic–pituitary–adrenal Axis
QR Code: Quick response code
SAMHSA: Substance abuse and mental health services association
SAM Axis: Sympathetic-adrenal-medullary Axis
TIC: Trauma informed care
US: United States
WHO: World Health Organization
